# Prediction of Multimorbidity in Brazil: Latest Fifth of a Century Population Study

**DOI:** 10.2196/44647

**Published:** 2023-05-30

**Authors:** Xi-liang Li, Hang Huang, Ying Lu, Randall S Stafford, Simone Maria Lima, Caroline Mota, Xin Shi

**Affiliations:** 1 School of Mathematics and Information Science Shandong Technology and Business University Yantai China; 2 School of Statistics Shandong Technology and Business University Yantai China; 3 Department of Biomedical Data Science School of Medicine Stanford University Stanford, CA United States; 4 Department of Medicine School of Medicine Stanford University Stanford, CA United States; 5 Management Engineering Department Universidade Federal de Pernambuco Recife Brazil; 6 Institute of Health Science China Medical University Shenyang China; 7 Manchester Metropolitan University Manchester Metropolitan University United Kingdom

**Keywords:** Brazil, demographic factors, logistic regression analysis, multimorbidity, nomogram prediction, prevalence

## Abstract

**Background:**

Multimorbidity is characterized by the co-occurrence of 2 or more chronic diseases and has been a focus of the health care sector and health policy makers due to its severe adverse effects.

**Objective:**

This paper aims to use the latest 2 decades of national health data in Brazil to analyze the effects of demographic factors and predict the impact of various risk factors on multimorbidity.

**Methods:**

Data analysis methods include descriptive analysis, logistic regression, and nomogram prediction. The study makes use of a set of national cross-sectional data with a sample size of 877,032. The study used data from 1998, 2003, and 2008 from the Brazilian National Household Sample Survey, and from 2013 and 2019 from the Brazilian National Health Survey. We developed a logistic regression model to assess the influence of risk factors on multimorbidity and predict the influence of the key risk factors in the future, based on the prevalence of multimorbidity in Brazil.

**Results:**

Overall, females were 1.7 times more likely to experience multimorbidity than males (odds ratio [OR] 1.72, 95% CI 1.69-1.74). The prevalence of multimorbidity among unemployed individuals was 1.5 times that of employed individuals (OR 1.51, 95% CI 1.49-1.53). Multimorbidity prevalence increased significantly with age. People over 60 years of age were about 20 times more likely to have multiple chronic diseases than those between 18 and 29 years of age (OR 19.6, 95% CI 19.15-20.07). The prevalence of multimorbidity in illiterate individuals was 1.2 times that in literate ones (OR 1.26, 95% CI 1.24-1.28). The subjective well-being of seniors without multimorbidity was 15 times that among people with multimorbidity (OR 15.29, 95% CI 14.97-15.63). Adults with multimorbidity were more than 1.5 times more likely to be hospitalized than those without (OR 1.53, 95% CI 1.50-1.56) and 1.9 times more likely need medical care (OR 1.94, 95% CI 1.91-1.97). These patterns were similar in all 5 cohort studies and remained stable for over 21 years. A nomogram model was used to predict multimorbidity prevalence under the influence of various risk factors. The prediction results were consistent with the effects of logistic regression; older age and poorer participant well-being had the strongest correlation with multimorbidity.

**Conclusions:**

Our study shows that multimorbidity prevalence varied little in the past 2 decades but varies widely across social groups. Identifying populations with higher rates of multimorbidity prevalence may improve policy making around multimorbidity prevention and management. The Brazilian government can create public health policies targeting these groups, and provide more medical treatment and health services to support and protect the multimorbidity population.

## Introduction

The co-occurrence of 2 or more chronic diseases in an individual is called multimorbidity [1,2]. Chronic diseases include hypertension, diabetes, cancer, cardiovascular disease, and chronic kidney disease [3]. Chronic diseases pose the greatest threat to human health in the modern world [4,5]. Chronic diseases usually occur together; this multimorbidity will cause greater harm to the body than any chronic disease on its own. The prevalence of multimorbidity has elevated significantly in recent years [6]. The health care needs of patients with multimorbidity differ from those of patients with a single condition and require a complex structured care plan for improved treatment [7]. This will be challenging in contexts where health care resources are scarce, and more medicines will be required to maintain the patient’s health. The vulnerability of these patients to safety issues is also elevated [8-10]. Increased prevalence of multimorbidity increases drug overconsumption, the complexity of disease management, the burden on health care services, and the rate of repeat hospitalizations, leading to increased health spending in the country [11,12]. An accurate estimation of multimorbidity prevalence is thus critical to assessing the public health impact of multimorbidity and predicting the medical needs of patients with multimorbidity [13].

The multimorbidity burden can be attributed to various causes, including sociodemographic and behavioral factors; gender is also associated with multimorbidity [14]. Countries differ in the ways they are affected by multimorbidity. Some developing countries do not have access to basic medical care or have sociocultural factors that affect some portions of the population. For example, women are socially, culturally, economically, and educationally disadvantaged in India, making them more vulnerable to chronic diseases [14-16]. In the United States, the prevalence of chronic disease in Black people is significantly higher than that in White people. However, some US studies suggest that gender is not a factor affecting multimorbidity [17,18]. As the population ages, the prevalence of multimorbidity gradually increases, because older adults are more susceptible to multimorbidity [19]. Although multiple studies have examined the relationship between sociodemographic factors and multimorbidity, few have explored the way these relationships change over time or attempted to predict future changes [20-22].

This study aimed to assess the dynamic distribution of 9 chronic diseases in the Brazilian population using cross-sectional survey data obtained from the Brazilian National Household Sample Survey (PNAD) and the Brazilian National Health Survey (PNS) in 1998, 2003, 2008, 2013, and 2019. PNAD and PNS are population-based surveys representing urban and rural Brazilians residing in private households, not in institutions. Data analysis was used to study whether sociodemographic factors, health care needs, and health insurance were associated with the prevalence of multimorbidity. Nomogram plots were used to predict the prevalence of multimorbidity in the future. Compared with traditional prediction methods, the use of nomograms provides excellent graphic visualization. The prediction model was validated in calibration curves by combining different influencing factors of participants. By predicting multimorbidity prevalence within groups, it may be possible to accurately identify the main affected population and ultimately reduce the burden on the medical system.

## Methods

### Data Availability

The following two data sources were combined in this study: (1) PNAD and (2) PNS data. The PNAD and PNS are complex multistage surveys conducted by the Brazilian Institute of Geography and Statistics to assess the situation of households in Brazil, and are available on the internet [23,24]. The PNAD study is a national survey. A 3-stage self-weighted cluster sampling technique was used. In the first stage, areas with larger populations or cities were selected. Other cities belonging to the same area were then divided into roughly the same level, and the selection was made through a system of size ratios. In the second stage, the contents of the 2010 census were systematically selected. The third stage identified households to survey on an area-specific basis [23]. PNS uses a 3-stage stratified sampling method. In the first stage, participants were selected by simple random sampling. The 2013 PNS study selection was based on households in the second stage, whereas the third stage involved a random selection of residents aged 18 years and above to answer the survey. The 2019 edition of the study involved individuals aged 15 years and above [24].

### Ethics Approval

The Brazilian Institute of Geography and Statistics received approval from the National Research Ethics Committee in Brazil and obtained consent from all PNAD and PNS participants. In case of nonagreement, the interviewees could always refuse to participate in the research. No additional data were collected for this study; we used only data presented by the Brazilian Institute of Geography and Statistics.

### Study Design

PNAD and PNS data were obtained from 5-year national surveys conducted in 1998, 2003, 2008, 2013, and 2019. Variables with more than 25% missing values were removed from our analysis to ensure the accuracy of the study. We included participants older than 18 years and excluded 6770 participants who did not answer all the questions about chronic diseases. The remaining 877,032 participants constitute a large pool of research participants.

In this study, binary logistic regression was used to compare multimorbidity group differences (gender, race, age, etc) and to calculate odds ratios (OR)—the ratio between the chance that an event occurs in 1 group and the chance that it occurs in another group. Prediction models can be used to aid health care providers’ decision-making by estimating the probability that a specific disease or condition is present (diagnostic models) or that a specific event will occur in the future (prognostic models). This model can be used to aid such efforts by predicting the strength of correlation between multimorbidity and other variables, as well as to determine if our logistic regression results are same with nomogram model, further confirming the consistency of all results in this study [25].

### Variables

This paper focuses on the prevalence of multimorbidity in Brazil. First, we selected 9 chronic diseases: back disease, rheumatism or arthritis, cancer, diabetes, asthma, hypertension, heart disease, chronic kidney disease, and depression. Chronic disease data are based on self-reported information in response to PNAD and PNS questions, such as “Has a physician or health care professional ever told you that you have diabetes?” or “Has any physician ever given you diabetes diagnosis?” Back diseases included chronic back problems, such as chronic back or neck pain, low back pain, sciatica, and vertebrae or disc problems, but the survey did not delimit the period of the pain occurrence [26]. Among the participants, 165,759 had 2 or more chronic diseases. Second, we selected 11 independent variables to assess the extent to which they were correlated with prevalence of multimorbidity: gender, race, age group, education, employment, region, health insurance, participant well-being, health service accessibility, health service need, and hospitalization. Third, we performed data analysis to estimate the future prevalence of multimorbidity under different influencing factors, such as gender and age. In the survey, subjective well-being was scored on a 5-point scale (1 being the best and 5 being the worst) based on participants’ perceptions their own health. The literacy levels of participants were assessed using literacy rate, and employment status was evaluated by asking whether they were employed during the survey period. This study made use of data on whether participants had been hospitalized within the past 12 months or had medical service needs within the past 2 weeks to analyze the effect of multimorbidity on public health, understand the use of medical services, and assist policy makers in formulating public health policies to reduce health spending. We divided the country into 5 regions (South, Southeast, Midwest, Northeast, and North) according to the official Brazilian territorial division to analyze the regional prevalence of frequently occurring diseases. This paper derives some new variables from existing data in databases, such as age group within official regions.

### Statistical Analysis

This study separately analyzed 3 aspects of multimorbidity separately: sociodemographic factors, health services, and subjective well-being. Descriptive analysis and data visualization were used to identify trends in changes among participants between years. The association between predictors and prevalence of multimorbidity was analyzed using hypothesis testing. Frequency analysis explored prevalence and the main prevalent population. The effect of variables on multimorbidity was studied using the logistic regression models. OR values were calculated with 95% CI. First, sociodemographic factors, including gender, race, age group, region, literacy, employment status, and health insurance status were considered to compute the probability of multimorbidity (with 1, without 0). Next, logistic regressions were used to investigate the prevalence of multimorbidity in participants with different levels of subjective well-being, health service availability, demand for health services, and hospitalization duration. This allowed us to analyze all the variables in the model independently to determine the OR of the predictor variables. Finally, a nomogram was developed to calculate risk scores using the model; these risk scores were then used to predict prevalence and determine the likelihood that individuals in certain sociodemographic categories will develop multimorbidity.

All statistical analyses were performed using SPSS Statistics, and R (version 4.0.5) was used to visualize the frequency analysis and model prediction.

## Results

### Descriptive Analysis

**Figure 1:**
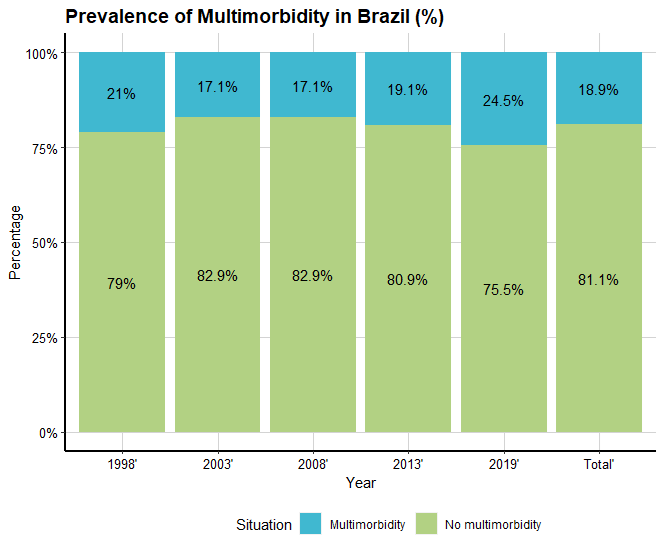
Prevalence of multimorbidity.

**Figure 2:**
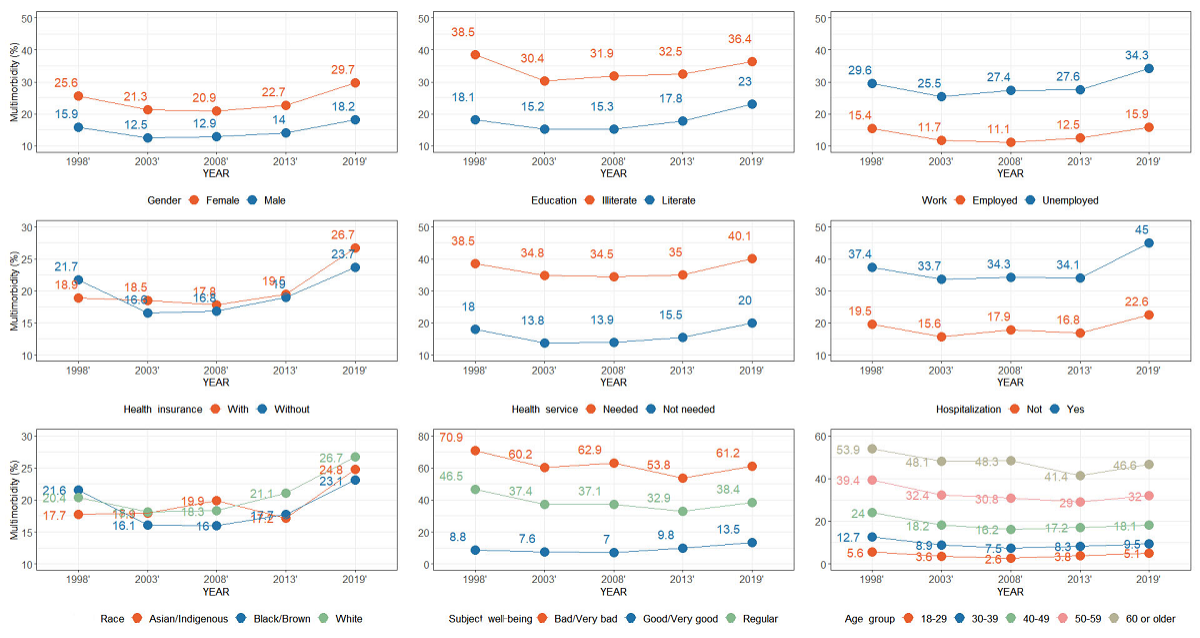
Percentage of multimorbidity by year with sociodemographic, subjective well-being, and health service characteristics.

A total of 877,032 participants were included in the analysis. Presence of multimorbidity was self-reported by participants aged 18 years or older. Data from 1998, 2003, 2008, 2013, and 2019 were used to analyze the association between 11 risk factors on the prevalence of multimorbidity. Total multimorbidity prevalence was 18.9%. An upward trend in multimorbidity prevalence was present in all years except for the 1998 to 2003 time period. The largest change occurred between 2013 and 2019, when prevalence increased by 5.4%, a significant change (Figure 1). We compared sociodemographic factors, subjective well-being, and health insurance status in different years to the prevalence of multimorbidity (Figure 2). Except for purchasing health insurance and ethnic factors, the change trends of other factors were the same in 5 years. The prevalence of multimorbidity in females was significantly higher than that in males and could reach 11.5% when influence of gender factors on the prevalence of multimorbidity. It was significantly less prevalent in literate than in illiterate participants, and in employed than in unemployed participants. The rates of health care use and hospitalization were significantly higher for participants with multimorbidity, and this difference became larger over time. Regarding self-assessment of health, people who felt their health was “bad” or “very bad” were more likely to have multimorbidity. The prevalence of people who have a better sense of well-being is rising, in contrast to the bad or very bad group. There is little difference in multimorbidity prevalence between people who have health insurance and those who do not, or between different racial or ethnic populations. However, the overall prevalence increased gradually, with the highest rate being seen in the most recent year of data (2019). In the study, the main incidence patterns of 9 diseases in 27 Brazilian federal units were studied. Hypertension and back disease are always the diseases with the highest prevalence in Brazil and are significantly more prevalent in all regions of Brazil.

### Logistic Regression and Model Prediction

Tables 1 and 2 indicate the relative degree of multimorbidity risk among participants from 1998 to 2019. Regression analysis revealed that in different cohorts, the changes in the risk of multimorbidity according to demographic variable did not change significantly. However, the magnitude of influence was consistent with the descriptive analysis. Among the differences found, the prevalence of multimorbidity in females was 1.72 times that in males (OR 1.72, 95% CI 1.69-1.74), consistent with the frequency analysis results in the 5-year cohort study. The Asian, Black or Brown, and White groups showed the same degree of prevalence of multimorbidity. The older the age, the higher the risk of multimorbidity. Participants aged 60 years and older were 20 times more likely than those aged 18 to 29 years to have multiple conditions (OR 19.6, 95% CI 19.15-20.07). In addition, being unemployed was positively associated with multimorbidity prevalence, and illiterate participants were 1.3 times more likely than literate participants to have multimorbidity (OR 1.26, 95% CI 1.24-1.28). Multimorbidity presence was higher in the Southern region (OR 1.31, 95% CI 1.28-1.34) than in the Northern region. Individuals having multimorbidity were mostly uninsured (OR 1.05, 95% CI 1.04-1.07).

**Table 1:** Results of the logistic regressiona examining the association of sociodemographic factors with multimorbidity (odds ratio [OR], 95% CI).

		1998	2003	2008	2013	2019	Overall
**Gender**							
	Male, OR (95% CI)	1.0 (reference)	1.0 (reference)	1.0 (reference)	1.0 (reference)	1.0 (reference)	1.0 (reference)
	Female, OR (95% CI)	1.76 (1.72-1.81)	1.76 (1.72-1.81)	1.60 (1.57-1.64)	1.76 (1.68-1.86)	1.89 (1.82-1.96)	1.72 (1.69-1.74)
	*P* value	<.001	<.001	<.001	<.001	<.001	<.001
**Race**							
	White, OR (95% CI)	1.0 (reference)	1.0 (reference)	1.0 (reference)	1.0 (reference)	1.0 (reference)	1.0 (reference)
	Asian/Indigenous, OR (95% CI)	0.73 (0.63-0.85)	0.89 (0.78-1.02)	1.03 (0.92-1.16)	0.91 (0.75-1.12)	1.06 (0.92-1.23)	0.90 (0.84-0.96)
	Brown or Black, OR (95% CI)	1.17 (1.14-1.20)	1.09 (1.06-1.12)	1.09 (1.06-1.11)	1.01 (0.96-1.06)	1.06 (1.02-1.10)	1.08 (1.07-1.09)
	*P* value	<.001	<.001	<.001	.289	.225	<.001
**Age group (years)**							
	18 to 29, OR (95% CI)	1.0 (reference)	1.0 (reference)	1.0 (reference)	1.0 (reference)	1.0 (reference)	1.0 (reference)
	30 to 39, OR (95% CI)	2.58 (2.48-2.69)	2.77 (2.64-2.90)	3.12 (2.96-3.29)	2.45 (2.17-2.75)	2.11 (1.91-2.31)	2.68 (2.62-2.75)
	40 to 49, OR (95% CI)	5.72 (5.49-5.95)	6.25 (5.98-6.53)	7.37 (7.02-7.74)	5.59 (5-6.26)	4.49 (4.10-4.90)	5.93 (5.79-6.07)
	50 to 59, OR (95% CI)	11.22 (10.76-11.70)	12.88 (12.32-13.47)	16.06 (15.30-16.86)	10.52 (9.42-11.76)	9.17 (8.41-10)	12 (11.71-12.28)
	60 or older, OR (95% CI)	17.91 (17.18-18.67)	21.71 (20.77-22.69)	28.11 (26.78-29.50)	15.45 (13.84-17.24)	14.46 (13.29-15.74)	19.60 (19.15-20.07)
	*P* value	<.001	<.001	<.001	<.001	<.001	<.001
**Education**							
	Literate, OR (95% CI)	1.0 (reference)	1.0 (reference)	1.0 (reference)	1.0 (reference)	1.0 (reference)	1.0 (reference)
	Illiterate, OR (95% CI)	1.37 (1.33-1.41)	1.17 (1.13-1.21)	1.16 (1.13-1.20)	1.09 (1.01-1.18)	1.04 (0.98-1.10)	1.26 (1.24-1.28)
	*P* value	<.001	<.001	<.001	<.001	<.001	<.001
**Work**							
	Employed, OR (95% CI)	1.0 (reference)	1.0 (reference)	1.0 (reference)	1.0 (reference)	1.0 (reference)	1.0 (reference)
	Unemployed, OR (95% CI)	1.38 (1.32-1.39)	1.51 (1.47-1.55)	1.66 (1.62-1.70)	1.51 (1.43-1.60)	1.58 (1.52-1.64)	1.51 (1.49-1.53)
	*P* value	<.001	<.001	<.001	<.001	<.001	<.001
**Region**							
	North, OR (95% CI)	1.0 (reference)	1.0 (reference)	1.0 (reference)	1.0 (reference)	1.0 (reference)	1.0 (reference)
	Northeast, OR (95% CI)	0.71 (0.68-0.75)	0.78 (0.75-0.82)	0.91 (0.88-0.95)	1.04 (0.97-1.21)	1.12 (1.06-1.18)	0.93 (0.91-0.95)
	Southeast, OR (95% CI)	0.66 (0.63-0.69)	0.93 (0.89-0.97)	1.13 (1.08-1.18)	1.14 (1.05-1.23)	1.31 (1.24-1.39)	1.05 (1.02-1.07)
	South, OR (95% CI)	0.83 (0.79-0.87	1.22 (1.16-1.28)	1.37 (1.31-1.44)	1.63 (1.49-1.79)	1.49 (1.40-1.60)	1.31 (1.28-1.34)
	Midwest, OR (95% CI)	0.86 (0.82-0.91)	1.15 (1.09-1.21)	1.21 (1.15-1.27)	1.27 (1.17-1.40)	1.19 (1.11-1.28)	1.19 (1.16-1.22)
	*P* value	<.001	<.001	<.001	<.001	<.001	<.001
**Health insurance**							
	With insurance, OR (95% CI)	1.0 (reference)	1.0 (reference)	1.0 (reference)	1.0 (reference)	1.0 (reference)	1.0 (reference)
	Without insurance, OR (95% CI)	1.25 (1.22-1.29)	1.0 (0.97-1.03)	1.03 (1-1.05)	1 (0.95-1.06)	0.90 (0.86-0.94)	1.05 (1.04-1.07)
	*P* value	<.001	<.001	<.001	<.001	<.001	<.001

^a^Binary logistic regression model by gender, race, age group, literacy, work, region, and health insurance.

**Table 2:** Results of the logistic regressiona examining the association of subjective well-being and use of health care with multimorbidity (odds ratio [OR], 95% CI).

		1998	2003	2008	2013	2019	Overall
**Subjective well-being**							
	Very good or good, OR (95% CI)	1.0 (reference)	1.0 (reference)	1.0 (reference)	1.0 (reference)	1.0 (reference)	1.0 (reference)
	Regular, OR (95% CI)	8.31 (8.10-8.52)	6.46 (6.30-6.62)	7.05 (6.88-7.22)	4.12 (3.92-4.34)	3.66 (3.53-3.80)	6.43 (6.35-6.51)
	Bad or very bad, OR (95% CI)	21.204 (20.28-22.17)	14.33 (13.75-14.94)	17.93 (17.23-18.66)	8.92 (8.25-9.66)	8.25 (7.77-8.77)	15.293 (14.97-15.63)
	*P* value	<.001	<.001	<.001	<.001	<.001	<.001
**Health** **service accessibility**							
	No, OR (95% CI)	1.0 (reference)	1.0 (reference)	1.0 (reference)	1.0 (reference)	1.0 (reference)	1.0 (reference)
	Yes, OR (95% CI)	1.19 (1.16-1.22)	1.39 (1.35-1.44)	1.45 (1.41-1.49)	1.46 (1.38-1.55)	1.33 (1.28-1.39)	1.32 (1.31-1.34)
	*P* value	<.001	<.001	<.001	<.001	<.001	<.001
**Health service need**							
	No, OR (95% CI)	1.0 (reference)	1.0 (reference)	1.0 (reference)	1.0 (reference)	1.0 (reference)	1.0 (reference)
	Yes, OR (95% CI)	1.68 (1.63-1.74)	2.03 (1.98-2.09)	1.99 (1.94-2.05)	2.10 (1.96-2.20)	2.03 (1.95-2.11)	1.94 (1.91-1.97)
	*P* value	<.001	<.001	<.001	<.001	<.001	<.001
**Hospitalization**							
	No, OR (95% CI)	1.0 (reference)	1.0 (reference)	1.0 (reference)	1.0 (reference)	1.0 (reference)	1.0 (reference)
	Yes, OR (95% CI)	1.35 (1.30-1.40)	1.52 (1.47-1.58)	1.57 (1.51-1.62)	1.54 (1.42-1.66)	1.86 (1.76-1.97)	1.53 (1.50-1.56)
	*P* value	<.001	<.001	<.001	<.001	<.001	<.001

^a^Binary logistic regression model by Subjective well-being, Health service accessibility, Health service need and Hospitalization, Yes: With multimorbidity, No: Without multimorbidity.

Table 2 shows a correlation of subjective well-being and medical assistance needs with multimorbidity. The correlation of “bad” and “very bad” subjective well-being with multimorbidity was 15 times (OR 15.29, 95% CI 14.97-15.63) that of “good” and “very good” in all cohorts of participants. Participants with multimorbidity were 1.3 times more likely to need health care services than those without multimorbidity (OR 1.32, 95% CI 1.31-1.34). In addition, patients with multiple diseases required 2 times more medical services (OR 1.94, 95% CI 1.91-1.97). Moreover, participants hospitalized during the study had a 1.5-fold prevalence of multimorbidity than those not hospitalized (OR 1.53, 95% CI 1.50-1.56).

**Figure 3:**
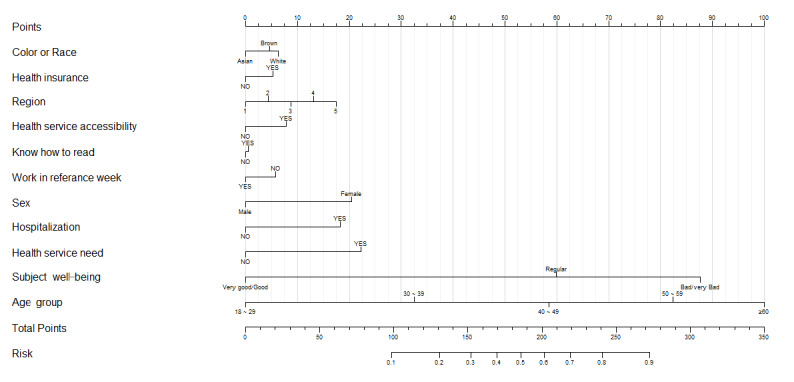
Nomograms using sociodemographic, health service, and subject-wellbeing factors selected for predicting multimorbidity risk. Region 1: North; Region 2: Northeast; Region 3: Southeast; Region 4: South; Region 5: Midwest. Given Points are independent points for each factor and Total Points are the sum of all points.

Nomograms have been developed as user-friendly prediction tools owing to their easy-to-interpret visualization interface. As shown in Figure 3, we constructed nomograms based on each category, with each variable exhibiting its axis corresponding to points representing their significance in the model. The corresponding points were summed up to obtain the total points, and the final probability was obtained by projecting the total points on the risk axis. The plot includes all significant variables based on logistic regression, incorporating sociodemographic factors, subjective well-being, and medical assistance within the nomogram. When calculating the scores for each risk factor, we found that risk scores were significantly higher for individuals aged 60 years or above, those with poorer subjective well-being, those in need of health services and hospitalization, and female and unemployed individuals. We analyzed the multimorbidity prevalence of these risk factors over 21 years to predict the likelihood of multimorbidity among members of these sociodemographic groups in the future. The prediction accuracy of the nomogram can be evaluated by the c-index value: a c-index value of 0.5-0.7 indicates poor prediction, whereas 0.7-0.9 indicates good prediction. The nomogram model developed in this study had a c-index value of 0.854, indicating that the model could correctly predict the results.

## Discussion

### Overview

Multimorbidity is a growing global challenge that places a strain on population health and imposes a large economic burden on society. Globally, multimorbidity is estimated to affect 65%-98% of those older than 65 years; the prevalence of multimorbidity is increasing, especially among older people, those with low language proficiency, and women [27,28]. Our findings indicate that the correlation of sociodemographic factors (specifically gender), health insurance status, and subjective well-being with multimorbidity was broadly consistent throughout a 21-year period. These findings are in line with those of other research. With aging, the prevalence of multimorbidity gradually increases, as reported in many studies [13,29,30]. Patients with multimorbidity take more medication, have more complicated disease management, use more health care services, and are more likely to undergo repeat hospitalizations, ultimately increasing overall national health expenditure [15,16].

From 1998 to 2019, the prevalence of multimorbidity first decreased, then increased. The decrease in prevalence could be because government expenditures on health care increased, which improved the health care system and reduced the rate of chronic disease. Particularly noteworthy is the change between 2013 and 2019, during which prevalence increased by 5.4%; in other time periods the increase was more gradual. Declining fertility has led to demographic changes in Brazil since the early 2000s, with an increase in the aging population, causing increased multimorbidity prevalence overall [31].

Studies in the United States show that Black people have a significantly higher rate of multimorbidity compared with White people [18]. Black or Brown populations in Brazil had lower incomes, less access to health insurance, and poorer living conditions [32], reflecting that living environment and income can be closely correlated with multimorbidity. This study examined the effect of educational attainment on multimorbidity, suggesting that illiterate adults had a higher prevalence of multimorbidity. Along with Brazil, this is the case in other countries, including India, South Africa, and Spain [33].

Subjective well-being was significantly related to prevalence of multimorbidity over the 21-year period analyzed in our study. In future research, targeted medical policies could be formulated for individuals with worse subjective well-being to reduce unnecessary medical expenditures [34].

Illness was assessed by whether or not the individual had purchased health insurance, although the time of purchase relative to disease occurrence is unknown. However, having a medical health plan increases access to medical services, reducing physical health risks [30].

In this study, only 19.1% of the participants had health insurance. Although some have chronic illnesses, they may be unaffordable for health insurance. In Brazil, patients have free access through the health care system to some essential medicines, but 1 research found low availability of drugs in all population strata. Drugs not provided by health care system can lead users to abandon prescribed treatments for not being able to buy them in the private sector with their own resources [35,36]. Previous studies have shown a gender difference in patterns of multimorbidity. A systematic review of most previous studies indicated that females had a greater prevalence of multimorbidity than males. This difference might be related to region, social, environmental, or economic factors. As these factors vary globally, their associations with multimorbidity might differ across populations [29,37,38].

Age has the most significant correlation with prevalence of multimorbidity, increasing significantly as the aging population increases. This has implications for countries, such as Brazil with aging populations. Subjective well-being also has a strong correlation with disease status, followed by health insurance status, hospitalization status, and gender. Based on the predicted situation, targeted medical policies should be implemented for individuals in groups with high rates of multimorbidity, reducing medical resource waste, and further improving the efficiency of medical services.

### Limitations

Our study analyzed the prevalence of multimorbidity in Brazil over the past 2 decades. Our study used self-reporting to determine whether participants had 1 or more chronic diseases; such self-reporting could be inaccurate if the participants did not remember their diagnoses, or the physician did not accurately diagnose a disease. Self-report has been widely used in public health and is considered a valid approach [31]. Another limitation is that some survey questions have changed over the course of the research. However, the content of the questions has not significantly changed; hence, these changes may not significantly affect the results. In addition, methodological modifications of data collection could have implications for calculating multimorbidity prevalence based on region.

### Conclusions

The distribution patterns of multimorbidity indicate that prevalence varies across social groups, but the differences remain largely consistent over the years in Brazil. This study also confirmed that the differences in demographic factors, including gender, education, employment status, and regional factors, significantly impair the public health situation in Brazil. The lack of change in multimorbidity throughout groups over the years offers an opportunity for epidemiologists and the public health sector to develop effective ways to prevent multimorbidity. Our findings may support the development of more effective public resource management. Among possible solutions, public health departments can provide more health services, particularly for groups with high prevalence of multimorbidity, such as women and older people. The government can also anticipate future patterns of multimorbidity and develop policies targeting individuals at high risk for multimorbidity and explore ways to reduce their risks. Additionally, by focusing on individuals in groups with the highest prevalence of multimorbidity, the government can implement public health policies to reduce the harm of multimorbidity while avoiding waste of public resources and assist the public health department in effectively dealing with the problems related to multimorbidity.

## Abbreviations

OR: odds ratio
PNAD: Brazilian National Household Sample Survey
PNS: Brazilian National Health Survey
